# What can we learn from general practitioners who left Spain? A mixed methods international study

**DOI:** 10.1186/s12960-023-00888-4

**Published:** 2024-01-23

**Authors:** Sara Calderón-Larrañaga, Ángel González-De-La-Fuente, Ana Belén Espinosa-González, Verónica Casado-Vicente, Óscar Brito-Fernandes, Niek Klazinga, Dionne Kringos

**Affiliations:** 1 Wolfson Institute of Population Health, Queen Mary University of London, London, United Kingdom; 2 XX Place Health Centre, Bromley By Bow Health Partnership, London, United Kingdom; 3 Global Business School for Health, University College London, London, United Kingdom; 4 Amsterdam UMC Location University of Amsterdam, Public and Occupational Health, Meibergdreef 9, Amsterdam, The Netherlands; 5 School of Public Health, Imperial College of London, London, United Kingdom; 6 Canberra Old Oak Surgery, London, United Kingdom; 7 Parquesol Health Centre, SACYL, Valladolid, Spain; 8 General Practice Teaching Unit, University of Valladolid, Valladolid, Spain; 9 Quality of Care, Amsterdam Public Health Research Institute, Amsterdam, The Netherlands

**Keywords:** Medical workforce migration, Medical workforce mobility, Medical workforce retention, General practice, Primary care

## Abstract

**Background:**

International mobility of health workforce affects the performance of health systems and has major relevance in human resources for health policy and planning. To date, there has been little research exploring the reasons why general practitioners (GPs) migrate. This mixed methods study aimed to investigate the reasons why Spain-trained GPs migrate and develop GP retention and recruitment health policy recommendations relevant to Spanish primary care.

**Methods:**

The study followed an explanatory sequential mixed methods study design combining surveys with semi-structured interviews and focus groups with GPs who qualified in Spain and were living overseas at the time of the study. The survey data examined the reasons why GPs left Spain and their intention to return and were analysed using quantitative methods. The transcripts from interviews and focus groups centred on GPs’ insights to enhance retention and recruitment in Spain and were analysed thematically.

**Results:**

The survey had 158 respondents with an estimated 25.4% response rate. Insufficient salary (75.3%), job insecurity and temporality (67.7%), excessive workload (67.7%), poor primary care governance (55.7%), lack of flexibility in the workplace (43.7%) and personal circumstances (43.7%) were the main reasons for leaving Spain. Almost half of the respondents (48.7%) would consider returning to Spanish general practice if their working conditions improved. Interviews and focus groups with respondents (*n* = 24) pointed towards the need to improve the quality of employment contracts, working conditions, opportunities for professional development, and governance in primary care for effective retention and recruitment.

**Conclusion:**

Efforts to improve GP retention and recruitment in Spain should focus on salary, job security, flexibility, protected workload, professional development, and governance. We draw ten GP retention and recruitment recommendations expected to inform urgent policy action to tackle existing and predicted GP shortages in Spanish primary care.

**Supplementary Information:**

The online version contains supplementary material available at 10.1186/s12960-023-00888-4.

## Introduction

The migration of health professionals has increased globally and within the European region over the last decades. The number of foreign-trained doctors working in OECD countries rose by 50% between 2006 and 2016 to nearly 500 000 [1]. Health professional mobility affects the performance of health systems in both sending and receiving countries, by aggravating or alleviating workforce shortages and regional maldistributions [2]. This holds significant implications within an international context marked by rising workforce constraints, especially in primary care, which have been exacerbated by the effects of the COVID-19 pandemic. These challenges pose a threat to the sustainability of healthcare systems, as emphasised by the timely editorial for the special collection on the medical workforce crisis in primary care in Europe, of which this study is part [3].

The existing literature on migration of health workers often relates to “*push and pull*” factors [4], which may represent the diametric conditions in relation to career prospects, income and working conditions available in the source and destination countries. Where the gap is marked, and hence health workers perceive this move can improve their professional and economic situation, the pull of the destination country takes effect [4]. However, most studies that investigate the migration of health professionals do not make distinctions among different specialities, such as primary care physicians or general practitioners (GPs) [5–8]. This knowledge gap is key in countries with primary care-based healthcare systems, particularly in cases like Spain, where diminished physician stock and projected workforce shortages disproportionately impact the primary care sector.

While the Spanish primary care is recognised as one of the best performing systems in Europe, it is currently facing unprecedented challenges [9]. Workforce shortages risk jeopardising its sustainability and performance, traditionally underpinned by robust foundations, such as free access at the point of delivery, comprehensive service coverage, multidisciplinary teams, and well-trained healthcare providers [9]. As per the Ministry of Health, the future balance between the supply and demand for medical professionals anticipates a 10% deficit in GPs over the next six years, potentially leading to a shortage of 10,000 GPs by 2028 [10]. This shortfall is attributed in part to increasing healthcare demands, an aging GP workforce (currently, 60% of GPs are aged 50 and older), and challenges in retaining doctors due to perceived difficult working conditions [10]. In this scenario, the migration of healthcare professionals further exacerbates the critical staff shortage issue. Over the past decade, a growing number of doctors trained in Spain have sought certification of suitability or good practice to work or study abroad, with about 12% being GPs [10, 11]. As per OECD data from 2020, sourced from destination country registries, approximately 5,176 doctors trained in Spain were registered overseas [12]. Of this count, an estimated 621 were GPs. This represents nearly 2% of Spain's total GP population, estimated at 39,666 in 2021 [12].

This study aimed to investigate the reasons why Spain-trained GPs who are currently practising abroad decided to leave the country and their intentions regarding returning. We also sought to explore their insights and recommendations for the development of effective retention and recruitment policies in the Spanish primary care by identifying the dimensions they value in their new work location. While the number of Spain-trained GPs practising overseas might represent a small proportion of the overall GP workforce, it has been argued to have major implications for what it represents—significant investments in human capital whose returns are realised abroad. Focusing research on this specific group may also yield valuable insights for addressing GP retention and recruitment challenges in a broader sense. It provides an opportunity to delve into the professional experiences of those who have worked in at least two different primary care systems, allowing for international comparisons and the identification of potential good practices relevant to the countries of origin, notably Spain [13].

## Methods

A mixed methods explanatory sequential study design was used, whereby survey data from self-administered online questionnaires informed semi-structured interviews and focus groups with GPs who qualified in Spain and were living overseas at the time of the study [14]. Data were collected and analysed between May and September 2022.

### Survey

The survey sought to investigate GPs’ reasons for migration and intention to return, and identify a sample of GPs who could be interviewed in a second stage in online focus groups and interviews. The preliminary questionnaire was developed in Spanish by the research team, informed by relevant theories and previous surveys used in health workforce migration, retention and recruitment literature [7, 8, 15–19]. Minor modifications to the survey design were made after piloting it with a small sample of GPs who qualified in Spain and were working overseas (*n* = 4). The characteristics of the questionnaire and dissemination strategy are specified in Box 1. The full questionnaire, including a translated English version, is provided in Additional file 1: Appendix S1.

Survey responses were categorised and included a “missing” value to allow a complete case analysis. Surveys with high levels of missingness (above 50% of sections not fulfilled) were excluded from the analysis. Data were described using percentages for categorical variables and median and interquartile range (IQR) for continuous variables. Associations between GPs’ sociodemographic characteristics (age, gender, nationality, country of work and exit year), reasons for leaving Spain and intention to return were assessed using univariate and multivariate logistic regression. The selection of covariates was based on prior subject knowledge from the scientific literature [4, 5, 7]. Regression analyses enabled deeper understanding of the motivations behind GP migration by exploring potential factors related to participants’ characteristics or the country of destination that could be associated with the reported reasons. Associations were considered statistically significant if the P-value was ≤ 0.05. All analyses were performed using STATA (version 17).

Box 1. Survey characteristics and dissemination strategy Characteristics of the survey:

The final questionnaire comprised a combination of open and closed-ended questions exploring:

1. key sociodemographic details (including age, gender, nationality, information on their GP qualification, country of work, exit year, current employment and working conditions);
2. reasons why respondents left the country where they originally qualified (Spain); and
3. their intention to return, explored through single response to mutually exclusive and complete options.

Survey dissemination strategy:

The survey was sent in electronic form following three consecutive dissemination strategies between May and September 2022:

1. The Spanish Organisation of Medical Colleges (OMC) forwarded the questionnaire to 650 GPs who had requested the certificate of suitability to work or study abroad after 2010. Two consecutive reminders were sent 10 days apart. The OMC could not contact all the GPs who had requested a certificate (5408) because most certificates missed an email address or had been processed by regional offices. The number of certificates of suitability does not reflect international mobility. The OMC may issue more than one certificate per doctor (their validity lasts 3 months) and many of those who request a certificate may not end up migrating. This explains the gap between the number of certificates issued by the OMC (5408) and the estimated total number of Spain-trained GPs working abroad as per OECD data (621).
2. The following national and international scientific and professional organisations relevant to general practice and primary care were reached for dissemination support:

- WONCA Europe, European General Practice Research Network (EGPRN), European Academy of Teachers in General Practice/Family Medicine (EURACT), European Forum of Primary Care (EFPC), Spanish Family Medicine Professional Societies (including, SemFYC, SEMERGEN, SEMG).

3. We advertised our study in social media and asked each respondent to forward the questionnaire to known eligible GPs.

### Interviews and focus groups

Twenty-four GPs who completed the questionnaire participated in either focus groups or semi-structured interviews intended to explore their recommendations for enhancing retention and recruitment in Spain. Focus groups enabled the generation of new insights by encouraging group interactions and discussions among Spain-trained GPs with diverse professional roles in various countries. Interviews, on the other hand, provided an opportunity to delve deeper into specific topics, such as GPs' engagement in teaching, training, or research. This blend of methods aimed to capture the depth of individual experiences and harness the synergy of group dynamics. In total, three semi-structured interviews and four focus groups (comprising 4 to 7 participants) were conducted in an alternating manner, scheduled based on participants’ availability. A stratified purposive sampling strategy was developed to obtain a range of views from GPs working in different countries, including those most represented in our survey (namely, United Kingdom, France, Ireland and Sweden) and holding various professional roles, such as clinical work, teaching, training, management or research. This allowed us to select information-rich cases while capturing variations across different participant characteristics. Sample size was determined iteratively guided by data saturation (the composition of focus groups and characteristics of participants are reported in Additional file 2: Appendix S2). Survey findings informed the development of the topic guide, which explored GPs’ professional experience in the country of destination and recommendations to inform GP retention and recruitment policies in Spanish primary care. A script summary is available in Box 2, while a more comprehensive version is provided in Additional file 3: Appendix S3 for further detail.

Interview transcripts and fieldnotes were analysed thematically. We combined a broadly deductive approach with a more inductive analysis guided by the framework proposed by Braun and Clarke [20]. Data were analysed as they were collected, which allowed for iterative modifications of the interview guide and the sampling strategy. We sought negative cases and discussed preliminary findings within the research team to enhance validity and rigour [21]. Data management was supported by NVivo V.10 software.

Box 2. Interview and focus group script summary

• **Introduction to the interview/focus group,** *mentioned that:*
– interviews were to be audio-recorded;
– how the recordings were to be used, transcribed, and stored;
– assurance of anonymity and confidentiality; and
– participants’ right to interrupt or withdraw from the interview.
• **Participants’ introduction,** *where they were asked to:*
– Introduce themselves, workplace, exit year, professional role.
– Highlight features they liked about the primary care/their job in the host country.
• **Interview** *covered the following thematic areas using questions aimed at clarifying what they found valuable in their current work locations, including instances of good practices that could be applied to the Spanish primary care context:*
– Job stability and flexibility
– Economic conditions: salary and pension
– Opportunities for professional development and research
– Healthcare management
– Prestige and recognition
– Training of GP trainees and medical students
• **Interview closure,** *included*:
– Brief summary
– Clarification questions

## Results

### Reasons for leaving and intention to return—survey findings

In total, 158 GPs completed the questionnaire, which represents a 25.4% response rate over the estimated eligible population (621, based on OECD statistics). No surveys were excluded as they all had a minimum of 50% of sections fulfilled. The median age of participants was 40 years (IQR 35–49) and 52% (*n* = 82) were female. 85% (*n* = 135) of those surveyed were Spanish, of which 9% (*n* = 15) had dual nationality (European or Latin American) (Table 1). The majority of respondents were early career clinicians (90% had qualified as GPs in the last 12 years) and considered leaving Spain after completing their GP training. There was a growing intention to migrate as the professional engagement in Spanish general practice increased (from undergraduate students to fully qualified GPs). Three-quarters (*n* = 119) of respondents had moved from Spain in the last 7 years, mainly to the United Kingdom (28%, *n* = 45), France (21%, *n* = 33), Ireland (18%, *n* = 29) or Sweden (11%, *n* = 18). Respondents’ professional role and employment are summarised in Additional file 4: Appendix S4: Table S1.

**Table 1:** Sociodemographic characteristics of survey respondents (*n* = 158)

Gender (%)	Female	82 (51.9)
	Male	63 (39.87)
	Non-binary	1 (0.63)
	Missing	12 (7.59)
Age (median, IQR)		40 (35–49)
Nationality (%)	Spanish	120 (75.95)
	Dual Spanish-European/Latin-American	15 (9.49)
	Other or non-available	23 (14.56)
Considered leaving Spain (%)	During medical degree	18 (11.39)
	During GP training	59 (37.34)
	After GP training	69 (43.67)
	Missing	12 (7.59)
Exit year (%)	< 2009	16 (10.13)
	2010–2014	23 (14.56)
	2015–2019	70 (44.3)
	> 2020	49 (31.01)
Country (%)	UK	45 (28.48)
	France	33 (20.89)
	Ireland	29 (18.35)
	Sweden	18 (11.39)
	Germany	6 (3.8)
	Switzerland	4 (2.53)
	Other*	23 (14.56)

*representation < 3: Portugal, Norway, Andorra, Belarus, Cameroon, Canada, Colombia, Arab Emirates, Luxemburg, The Netherlands, Poland, USA, Italy, Denmark

**Fig. 1:**
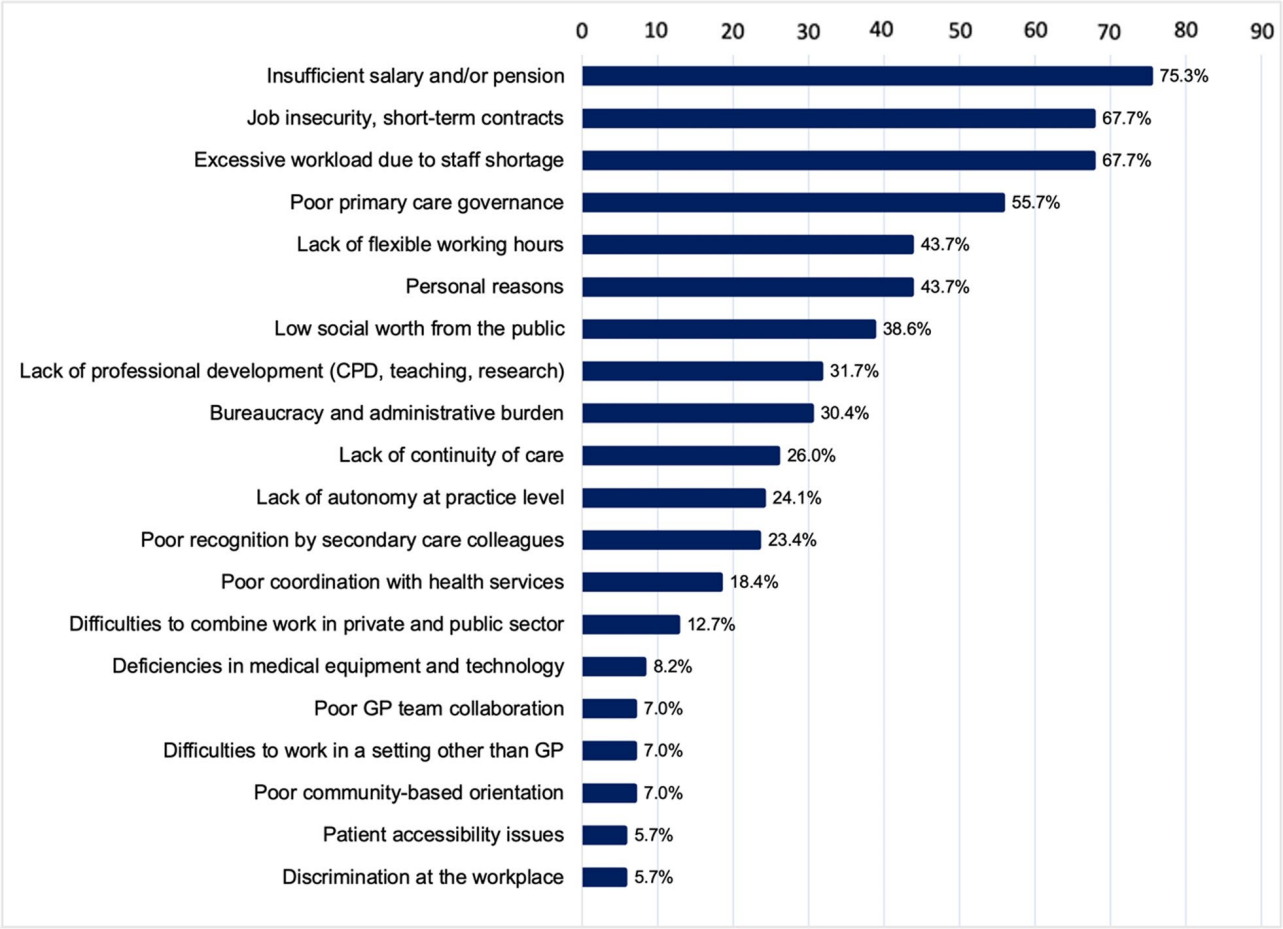
Main reasons for leaving Spanish general practice (*n* = 158)

Most participants (93%, *n* = 147) reported more than one reason for leaving Spain. Salary (including lack of remuneration for certain professional activities, such as teaching, research or management), employment insecurity and temporality, excessive workload, poor primary care governance, lack of flexibility in the workplace, as well as personal reasons were the most reported “*push factors*” (Fig. 1). The multivariate analyses concerning the association between the five main reasons for leaving Spain reported in the questionnaire and patients’ sociodemographic characteristics are available in Table 2. Briefly, younger age was significantly associated with reporting job insecurity (crude OR 0.94 [95% CI 0.90–0.98]) and lack of flexible working hours (crude OR 0.96 [95% CI 0.92–0.99]) as the main reasons for leaving. GPs who left Spain in the last 7 years reported less satisfaction with salary (crude OR 3.41 [95% CI 1.56–7.46]), workload (crude OR 2.23 [95% CI 1.06–4.71]) and primary care governance (crude OR 2.20 [ 95%CI 1.05–4.49]) compared to GPs who migrated before 2015. Those who moved to UK and Sweden were more likely to leave Spain due to lack of flexibility (crude OR 2.63 [95%CI 1.02–6.77] and 3.61 [95%CI 1.08–12.05], respectively), while GPs in France and Ireland were more likely to report financial reasons (crude OR 3.64 [95%CI 1.12–11.85] and 5.63 [95%CI 1.41–22.48], respectively). Approximately half of the respondents (49%, *n* = 77) would return to Spain if working conditions improved (Additional file 4: Appendix S4, table S2). The intention to return was not significantly associated with the sociodemographic characteristics of respondents (Additional file 4: Appendix S4, Tables S3).

**Table 2:** Association between main reasons for leaving Spain and respondents’ sociodemographic characteristics (n = 158)

		Financial			Lack of stability			Workload			Poor governance			Lack of flexibility		
		N 119, 75.3%	OR (95% CI)	OR (95% CI)*	N 107, 67.7%	OR (95% CI)	OR (95% CI)*	N 107, 67.7%	OR (95% CI)	OR (95% CI)*	N 88, 55.7%	OR (95% CI)	OR (95% CI)*	N 69, 43.7%	OR (95% CI)	OR (95% CI)*
Gender	Female	58 (70.7)	0.56 (0.20–1.04)	0.45 (0.18–1.14)	61 (74.4)	1.67 (0.82–3.41)	1.47 (0.67–3.23)	55 (67.1)	0.75 (0.37–1.55)	0.73 (0.34–1.57)	43 (52.4)	0.59 (0.30–1.16)	0.60 (0.29–1.22)	38 (46.3)	0.95 (0.49–1.83)	0.82 (0.41–1.66)
	Male	53 (84.1)	1	1	40 (63.5)	1	1	46 (73)	1	1	41 (65.1)	1	1	30 (47.6)	1	1
	Non-binary	1 (100)			0			0			0			0		
	Missing	7 (58.3)			6 (50)			6 (50)			4 (33.3)			1 (8.3)		
Age (median, IQR)		40 (35,51)	1.04 (0.99–1.09)	**1.07 (1.01–1.15)**	38 (34,45)	**0.94 (0.90–0.98)**	**0.95 (0.90–0.99)**	39 (35,49)	0.99 (0.95–1.03)	1.01 (0.96–1.06)	39.5 (35,51)	1.01 (0.97–1.05)	1.02 (0.97–1.07)	37 (35,45)	**0.96 (0.92–0.99)**	0.96 (0.92–1.01)
Nationality	Spanish	94 (78.3)	2.07 (0.56–7.60)	2.40 (0.55–10.40)	81 (67.5)	1.19 (0.33–4.30)	1.43 (0.35–5.80)	84 (70)	1.33 (0.37–4.84)	1.99 (0.50–8.02)	72 (60)	2.63 (0.73–9.46)	2.63 (0.68–10.12)	56 (46.7)	1.05 (0.30–3.63)	1.08 (0.29–4.00)
	Spanish & other	11 (73.3)	1.57 (0.29–8.42)	2.16 (0.31–15.24)	13 (86.7)	3.71 (0.54–25.59)	5.17 (0.66–40.56)	10 (66.7)	1.14 (0.22–5.84)	1.67 (0.28–9.85)	8 (53.3)	2.00 (0.41–9.84)	2.11 (0.39–11.54)	7 (46.7)	1.05 (0.22–5.00)	1.27 (0.24–6.82)
	Other/non-available	14 (60.9)	1	1	13 (56.5)	1	1	13 (56.5)	1	1	8 (34.8)	1	1	6 (26.1)	1	1
Country	UK	31 (68.9)	1.44 (0.56–3.69)	2.75 (0.86–8.76)	36 (80)	2.00 (0.72–5.59)	**3.43 (1.00–11.76)**	25 (55.6)	0.46 (0.18–1.23)	0.56 (0.19–1.66)	26 (57.8)	1.64 (0.66–4.06)	2.10 (0.75–5.91)	24 (53.3)	**2.63 (1.02–6.77)**	**2.98 (1.04–8.50)**
	France	28 (84.9)	**3.64 (1.12–11.85)**	2.40 (0.63–9.20)	20 (60.6)	0.77 (0.28–2.20)	1.21 (0.38–3.84)	25 (75.8)	1.17 (0.28–3.54)	0.99 (0.29–3.34)	21 (63.6)	2.10 (0.78–5.63)	1.69 (0.56–5.08)	13 (38.4)	1.50 (0.54–4.14)	1.46 (0.48–4.41)
	Ireland	26 (69.7)	**5.63 (1.41–22.48)**	**6.97 (1.22–39.62)**	15 (51.7)	0.54 (0.19–1.50)	0.88 (0.26–2.97)	19 (65.5)	0.71 (0.24–2.10)	0.70 (0.21–2.41)	16 (55.2)	1.48 (0.54–4.02)	1.58 (0.50–5.01)	11 (37.9)	1.41 (0.49–4.04)	1.49 (0.47–4.77)
	Sweden	14 (77.8)	2.28 (0.61–8.45)	4.39 (0.87–22.08)	14 (77.8)	1.75 (0.46–6.59)	3.80 (0.68–21.14)	14 (77.8)	1.31 (0.34–5.06)	2.79 (0.50–15.74)	10 (55.6)	1.50 (0.47–4.76)	2.23 (0.60–8.30)	11 (61.1)	**3.61 (1.08–12.05)**	**4.03 (1.06–15.24)**
	Other	20 (60.6)	1	1	22 (66.7)	1	1	24 (72.7)	1	1	15 (45.5)	1	1	10 (30.3)	1	1
Exit year	Before 2015	22 (56.4)	1	1	24 (61.5)	1	1	21 (53.9)	1	1	16 (41)	1	1	15 (38.5)	1	1
	After 2015	97 (81.5)	**3.41 (1.56–7.46)**	**7.52 (2.35–24.09)**	83 (69.8)	1.44 (0.68–3.06)	1.72 (0.60–4.92)	86 (72.3)	**2.23 (1.06–4.71)**	2.31 (0.88–6.06)	72 (60.5)	**2.20 (1.05–4.49)**	**2.66 (1.04–6.85)**	54 (45.4)	1.33 (0.63–2.78)	1.27 (0.50–3.26)

In bold statistically significant findings

*OR adjusted by the remaining sociodemographic variables included in the table

### What do GPs value? Recommendations to improve retention and recruitment in Spain—interview and focus group findings

Interviews and focus groups with a diverse sample of Spain-trained GPs led to the identification of the following four dimensions and the ten recommendations summarised in box 3 relevant to improving GP retention and recruitment in Spain:

#### Employment contracts: stability, flexibility and salary

Job stability (often ensured through retention strategies to remainlinked to the same practice or locality) was believed to enable improved patient care, workplace integration and prospects for personal and professional development, as noted by one participant: *“In order to do a good job, you need stability. I mean, you cannot provide continuity of care to a patient if you keep moving from one place to another” (GP B1.3 in UK).* GPs also valued being able to “*choose*” their working conditions, including where, when, and how often they were meant to practise. Flexibility proved key for adapting job contracts to GPs’ specific and changing professional and personal circumstances, including the possibility to combine clinical practice with other professional interests, such as research, management, teaching or training. Salary was also highlighted as a relevant incentive. GPs valued not having to work night shifts or extra hours to reach a satisfactory income, and being reimbursed for additional activities, such as teaching, specific procedures and research.

#### Work conditions: time allocation and comprehensiveness

Sufficient time to listen to and deal with patients’ concerns was believed to contribute to good practice and greater work satisfaction: *"from seeing 40, 50 patients daily, here I’m seeing 15… each patient has a minimum of 15 min or sometimes even half an hour"* (GP B2.4 in Ireland). GPs also valued having protected time in their schedule for non-patient-facing roles and tasks, including teaching, training or administrative work (e.g., processing referrals, test results and repeat prescriptions): “*we don’t do anything that is not included in the diary*” (GP B5.1 in Sweden). Greater capacity to respond to patients’ needs in primary care was also highlighted, which was often achieved by: (1) expanding multi-professional teamwork in general practice (including pharmacists, physiotherapists, and social prescribers) and upskilling practice staff; (2) ensuring access to a broad range of diagnostic tests and treatments with clear and  consistent guidelines regarding the referral process; and (3) strengthening communication channels with secondary care (e.g., regular case discussion meetings, direct dialling).

#### Professional development: training, teaching, and research

Being provided with (and required to engage in) continuing professional development was considered key to ensuring competency and care quality. Beyond the formal training, GPs also called attention to the importance of peer education networks to reflect on daily practice and mentoring programmes for guidance on how to advance their professional interests: *“The opportunity to sit down with one of your colleagues who has more experience, […] tell him what's going on with you, how you see yourself professionally, where you want to go”* (GP B1.2 in UK). The involvement of GPs in teaching fundamental clinical skills throughout the medical degree was believed to grant prestige and reputation to the profession. Formal training and networking opportunities to support GP trainees and professors were also valued. According to the academic GPs interviewed, the presence of primary care university departments, strengthened links between universities and GP practices, and specific funding streams aimed at primary care clinicians fostered research. Ensuring that academic work was both compensated and carried out within their regular work hours (instead of in their spare time) was also keyfor their involvement and for creating opportunities to effectively combine academic and clinical roles.

#### Leadership and management capacity

GPs valued having certain degree of operational autonomy at practice level to manage the internal organisation, including decisions concerning the distribution of tasks across staff members and prioritisation of specific programmes or interventions. This was believed to enhance suitability to local contingencies and foster a sense of ownership and greater involvement: *“having decision-making power over my work, over how we want to do it, the philosophy of the health centre, […] all of this is essential to me. I can't give that up anymore”* (GP B3.4 in Belgium). Opportunities to become involved in higher-level management, including dual clinical and managerial roles, were also valued and believed to contribute to management practices that were more accountable and responsive to practitioners’ demands. The importance of strong representative bodies (such as, royal colleges, scientific societies, medical committees) in advocating for general practice, training, and research was also highlighted. GPs emphasised the potential of these organisations to foster public awareness and social worth, and to inform or question health policy in GPs’ best interest.

## Discussion

### Summary

Using an explanatory sequential mixed methods design, we identified the main reasons why Spain-trained GPs left the country and their recommendations to enhance GP retention and recruitment in Spanish primary care. Based on 158 respondents, our survey revealed relative pay, employment insecurity and temporality, excessive workload, poor primary care governance, lack of flexibility in the workplace and personal reasons as main contributors. Importantly, almost half of the respondents would consider returning to Spanish general practice if their professional demands were satisfied. Interviews and focus groups with a diverse sample of 24 Spain-trained GPs pointed towards the need to improve the quality of employment contracts, working conditions, opportunities for professional development, and governance in primary care for effective retention and recruitment.

### Comparison with existing literature

Our study confirmed that economic factors, specifically the prospect of financial gain, motivate mobility [5, 7, 22, 23]. In Spain, the salary of GPs (adjusted for purchasing power parity) is below the European (19 EU) and OECD averages, including the host countries most represented in our survey, namely United Kingdom, France, Ireland and Sweden [24, 25]. In addition, GP compensation in Spain shows a small margin for growth with additional professional responsibilities, financial incentives or seniority, which may also contribute to explaining our findings [24].

Consistent with previous research, job security and flexibility acted as influential “push” and “pull” factors for migration [26]. Our study supports urgent policy action to tackle job insecurity in Spain, especially in a context where 37.5% of doctors have a temporary employment contract (of which 40.9% are in the 40–60 age group) according to recent literature [27]. In addition to job security, work contracts from Spanish regional authorities offer GPs limited flexibility in tailoring their schedules to personal and professional needs, impacting job satisfaction and opportunities to engage in additional professional roles [9]. Our study underscores the need for policy measures to enhance flexibility for improved job satisfaction, professional advancement, and retention.

Excessive workload has also been found in the literature to trigger physicians’ mobility and GPs leaving their profession early [16, 28, 29]. GPs in Spain spend less time with their patients compared to other European countries (average consultation length is 7.8 min) and face a high workload (35.1% of GPs see between 36 and 45 patients daily), which may contribute to explaining poor job satisfaction and GP retention issues observed in our study [27, 30]. Interviewees also highlighted the need to strengthen coordination with secondary care, broaden access to diagnostics and treatments, and ensure multidisciplinary general practice teams as a means of enhancing primary care comprehensiveness and job satisfaction, which has also been reported elsewhere [17, 31–33].

Strengthened career and professional prospects as clinicians, trainers, mentors or researchers have also been found to attract international physicians and retain GPs [34]. Our study contributed to knowledge by identifying relevant opportunities for continuing professional development, including GP accreditation and revalidation schemes (which is common practice in many European countries [35]), peer-led networks, as well as targeted support and incentives. According to the Spanish Organisation of Medical Colleges, almost half of the doctors in Spain do not receive any additional remuneration or workload adjustment for training students or GPs [27]. Similarly, there are no primary care university departments nor specific (or accessible) research funding schemes aimed at GPs, which may contribute to explaining observed retention issues [36].

In relation to leadership and management, our study findings seem to align with “*the principle of minimum specification*”, whereby practitioners are allowed the autonomy to respond to local contingencies and decide *how* to deliver services on an agreed broad goal and principle, as a way of ensuring efficient and effective healthcare provision [37]. In line with published literature, our study found that strong professional organisations seem to play an important role in sharing critical resources and productively participating in regulatory processes in GPs’ best interest [38].

### Strengths and limitations

To our knowledge, this is the first study investigating the reasons why Spain-trained GPs migrate. While many studies have investigated the migration of physicians, there is a dearth of research that delves into the migration of primary care practitioners. Our survey could potentially serve as a valuable resource for subsequent studies exploring this subject elsewhere, provided that it is suitably adapted to other languages and country-specific contexts. Moreover, our explanatory mixed methods design allowed to gain deep understanding on perceived challenges and opportunities, and develop evidence-based GP retention and recruitment policy recommendations based on GPs’ professional experience (box 3), which are expected to address the workforce crisis in Spain.

The study had limitations. As explained in box 1, the total number GPs who had requested a certificate of suitability to work abroad could not be contacted, which might have led to selection bias and affected the representativeness of the sample and generalisability of the results. Lack of available data on the distribution of Spain-trained GPs in different OECD countries and their sociodemographic characteristics meant that it was not possible to confirm or assess this.

### Implications for research and practice

Building on research findings, we have developed 10 evidence-based health policy recommendations to enhance GP retention and recruitment in Spanish primary care (summarised in Box 3). Our findings and recommendations are anticipated to hold significant value and applicability elsewhere in Europe given the shared context of primary care workforce crisis and the growing international interconnectedness of healthcare workforce policy [3]. Our study also contributes to framing health workforce international mobility as an opportunity to share examples of good practice and suggestions for improvement, while instigating policy learning and transfer across Europe. Study findings are expected to inform urgent policy action to tackle existing and predicted GP shortages, which risk jeopardising the sustainability of primary care [39, 40].

Box 3. Policy recommendations to enhance GP retention and recruitment in Spanish primary care

1. **Create** working conditions that ensure job stability, sufficient salary, flexibility, and a healthy work–life balance
2. **Protect** workload by also acknowledging non-patient facing activities (e.g., follow-up tasks, administrative work, management, teaching, research, training)
3. **Maximise** the potential of each professional within multidisciplinary general practice teams
4. **Enhance** direct and protocolised access to diagnostics and treatments from primary care
5. **Strengthen** communication channels with secondary care (e.g., regular case discussion meetings, direct dialling)
6. **Promote** professional development through access to mentoring, peer-led networks, continuous professional development opportunities, attractive career prospects and revalidation
7. **Align** medical education with health service requirements by increasing general practice involvement in undergraduate training
8. **Develop** an academic portfolio with specific funding schemes, primary care departments in medical schools and training opportunities
9. **Build** leadership and management capacity for primary care governance and planning
10. **Support** organisations representing the interests of general practice and primary care

## Conclusions

Our mixed methods study identified salary, job security, flexibility, workload, professional development, and governance as the main contributors to international mobility of Spain-trained GPs. The finding that nearly half of the respondents would be willing to return to Spanish general practice if their professional conditions improved highlights the great potential of the proposed health policy recommendations to enhance retention in a context marked by substantial existing and projected shortages of GPs. Further research will be critical to evaluate the impact of these policies on the overall retention and stability of the general practice workforce in Spanish primary care.

### Supplementary Information

**Additional file 1. **Questionnaire (in Spanish).

**Additional file 2.** Qualitative sample characteristics.

**Additional file 3.** Focus group/Semi-structured Interview Script.

**Additional file 4.** Survey results.

## Data Availability

The datasets used and/or analysed during the current study are available from the corresponding author on reasonable request.

## Abbreviations

GPs: General practitioners
OMC: Organisation of Medical Colleges
IQR: Interquartile range
OR: Odds ratio
