# Supplementary material for: What can we learn from general practitioners who left Spain? A mixed methods international study
Source: Hum Resour Health. 2024 Jan 23;22:9. doi: 10.1186/s12960-023-00888-4 (PMC10804741; doi:10.1186/s12960-023-00888-4)
Supplement: Supplementary file 1 — Additional file 1. Questionnaire (in Spanish). [file 12960_2023_888_MOESM1_ESM.docx]

**Additional file 1: Appendix S1. Questionnaire (in Spanish)**

Gracias por compartir su experiencia y opinión en este estudio sobre especialistas en Medicina Familiar y Comunitaria (MFyC) cualificados en España que deciden emigrar al extranjero.

Los datos muestran que el sistema sanitario español ha experimentado una salida sostenida de profesionales desde 2010, de los cuales la mayoría son médicos/as de familia. Sin embargo, las razones por las cuales los/as especialistas en MFyC abandonan nuestro país, a qué se dedican ahora, o las barreras que dificultan un posible regreso no están claras. Este estudio, financiado por el Instituto de Investigación en Salud Pública de Ámsterdam UMC en colaboración con la Organización Médica Colegial y médicos/as de familia e investigadores españoles, pretende responder a estas preguntas. Confiamos en que los resultados contribuyan a mejorar las oportunidades y condiciones de trabajo de médicos/as de familia en España.

Para ello, le agradeceríamos que respondiera al siguiente cuestionario. Se le harán preguntas sobre las razones por las que decidió emigrar, los factores que influyeron en la elección del país de destino y posibles barreras para regresar a España. Se tarda aproximadamente **25 minutos** en completarlo.

Queremos recordarle que su participación es voluntaria y respeta el anonimato. Sus respuestas serán analizadas por el equipo investigador de Ámsterdam UMC, y reportadas de forma agregada. Puede facilitarnos sus datos de contacto al final de la encuesta si desea participar en una entrevista grupal, parte del mismo estudio. En tal caso, sus respuestas y participación permanecerán confidenciales. El estudio ha obtenido la aprobación ética en Ámsterdam UMC y cumple con el Reglamento General de Protección de Datos 2016/679.

Puede ponerse en contacto con el quipo investigador dirigiéndose a Sara Calderón-Larrañaga en [s.calderon@qmul.ac.uk](mailto:s.calderon@qmul.ac.uk) si tiene cualquier pregunta o desea más información.

1. Preguntas filtro

¿Completó la especialidad de Medicina Familiar y Comunitaria (MFYC) en España?

| O | No [fin del cuestionario. Mensaje automático: Lamentamos comunicarle que no es elegible para este estudio, pues está únicamente dirigido a médicos/as de familia especializados/as en España] |
| --- | --- |
| O | Sí [sólo si responden sí pueden continuar] |

¿Vive o trabaja actualmente en el extranjero?

| O | No [fin del cuestionario. Mensaje automático: Lamentamos comunicarle que no es elegible para este estudio, pues está únicamente dirigido a médicos/as de familia que viven o trabajan en el extranjero] |
| --- | --- |
| O | Sí [sólo si responden sí pueden continuar] |

¿En qué país trabaja actualmente?

| País: |
| --- |

¿En qué año emigró de España?

| Año: |
| --- |

1. **¿Qué le hizo emigrar de España?** Por favor, **marque todas las opciones** que se correspondan con usted:

|  | **Se corresponde conmigo** |
| --- | --- |
| - [2.1] Motivos personales (ej. mejores oportunidades laborales para mi pareja, aprender sobre una nueva cultura, sociedad, mejorar mis competencias lingüísticas) | O |
| - [2.2] Falta de oportunidades para realizar una tesis doctoral o investigar (ej. ausencia de departamentos universitarios, infraestructura, financiación) | O |
| - [2.3] Carencias en la formación médica continuada y/o falta de oportunidades para una mayor especialización | O |
| - [2.4] Falta de oportunidades para la docencia de estudiantes de medicina o residentes, y/o de combinarlo con la clínica de forma satisfactoria | O |
| - [2.5] Falta de oportunidades para involucrarme en la gestión sanitaria | O |
| - [2.6] Falta de oportunidades para trabajar en un ámbito asistencial diferente a la atención primaria (ej. urgencias (extra)hospitalarias, paliativos, etc.) | O |
| - [2.7] Salario y/o pensión insuficientes | O |
| - [2.8] Contratos de corta duración o poca estabilidad laboral y económica | O |
| - [2.9] Ausencia de remuneración por actividades profesionales adicionales (ej. horas extras, investigación, docencia) | O |
| - [2.10] Falta de oportunidades para combinar la asistencia en el sistema público y el sector privado | O |
| - [2.11] Excesiva carga de trabajo en atención primaria debido a la falta de personal | O |
| - [2.12] Excesiva burocracia y trabajo administrativo | O |
| - [2.13] Falta de un horario de trabajo flexible (ej. para conciliar, combinarlo con la investigación, docencia u otros intereses personales o profesionales) | O |
| - [2.14] Deficiencias en equipamiento médico o tecnológico (incluida la historia clínica electrónica) | O |
| - [2.15] Falta de autonomía a nivel de centro de salud para implementar innovaciones (ej. cambios organizativos, contratación de servicios adicionales) | O |
| - [2.16] Insatisfacción con la gestión del sistema de atención primaria | O |
| - [2.17] Problemas de accesibilidad para los/as pacientes | O |
| - [2.18] Imposibilidad de tener un cupo de pacientes asignado (continuidad asistencial o longitudinalidad) | O |
| - [2.19] Mala coordinación con los servicios de salud comunitarios y/o hospitalarios (incluido urgencias, salud mental, especialistas hospitalarios) | O |
| - [2.20] Mala coordinación con salud pública | O |
| - [2.21] Escasa colaboración con el equipo del centro de salud (ej. personal de enfermería, auxiliar, administrativo) | O |
| - [2.22] Falta de oportunidades para participar en actividades comunitarias de promoción de la salud | O |
| - [2.23] Discriminación en el lugar de trabajo por motivos de género, identidad u orientación sexual, raza, religión, diversidad funcional u otros. | O |
| - [2.24] Escaso reconocimiento por parte de los colegas de profesión | O |
| - [2.25] Escaso prestigio social por parte de la población general | O |
| - [2.26] Otro motivos: | O |

1. **Seleccione los 3 principales motivos por lo que decidió emigrar de España en orden de importancia (**en el desplegable solo le aparecerán las opciones que previamente ha seleccionado):

Primer motivo[ ]

Segundo motivo[ ]

Tercer motivo [ ]

1. ¿**Qué le hace permanecer** en el país donde actualmente vive y trabaja? Por favor, **marque todas las opciones** que se correspondan con su situación:

|  | **Se corresponde conmigo** |
| --- | --- |
| - [4.1] Motivos personales (ej. mejores oportunidades para hijos/as o pareja, vínculos con la familia, amistades y/o la sociedad en el país de destino) | O |
| - [4.2] Instalaciones, entorno y financiación adecuadas para la investigación | O |
| - [4.3] Acceso a una formación médica continuada de alta calidad y oportunidades para una mayor especialización | O |
| - [4.4] Vínculos establecidos con facultades de medicina y/o programas de especialidad, pudiendo combinar la docencia con la práctica clínica | O |
| - [4.5] Posibilidades de liderazgo y/o de involucrarme en la gestión sanitaria | O |
| - [4.6] Posibilidades de trabajar en otro ámbito asistencial diferente a la atención primaria (ej. urgencias (extra)hospitalarias, paliativos, etc.) |  |
| - [4.7] Mayor salario y/o pensión | O |
| - [4.8] Contratos de larga duración o mayor estabilidad laboral y económica | O |
| - [4.9] Posibilidad de aumentar y diversificar los ingresos a través de responsabilidades adicionales (ej. horario ampliado, sustituciones, gestión sanitaria, formación, docencia y/o investigación) | O |
| - [4.10] Posibilidad de combinar la asistencia en el sistema público y el sector privado | O |
| - [4.11] Tiempo suficiente para los/as pacientes | O |
| - [4.12] Menos burocracia y trabajo administrativo | O |
| - [4.13] Horario de trabajo flexible (ej. para conciliar, combinarlo con la investigación, docencia u otros intereses personales o profesionales) | O |
| - [4.14] Mejor equipamiento médico y tecnológico (incluida la historia clínica electrónica) | O |
| - [4.15] Mayor autonomía a nivel de centro de salud para implementar innovaciones (e.j., cambios organizativos, contratación de servicios adicionales) | O |
| - [4.16] Mejor gestión del sistema de atención primaria, incluida una mayor respuesta a las demandas y necesidades de los/as profesionales | O |
| - [4.17] Mayor accesibilidad para los/as pacientes | O |
| - [4.18] Posibilidad de tener un cupo de pacientes asignado (mayor continuidad asistencial o longitudinalidad) | O |
| - [4.19] Buena coordinación con los servicios de salud comunitarios y/o hospitalarios (incluido urgencias, salud mental, especialistas hospitalarios) | O |
| - [2.20] Buena coordinación con salud pública | O |
| - [2.21] Buena colaboración con el equipo del centro de salud (ej. personal de enfermería, auxiliar, administrativo) | O |
| - [2.22] Oportunidades para participar en actividades comunitarias de promoción de la salud | O |
| - [3.23] Ambiente laboral inclusivo y respetuoso con la diversidad | O |
| - [3.24] Mayor reconocimiento entre colegas de profesión | O |
| - [3.25] Mayor prestigio social por ser médico/a de familia | O |
| - [3.26] Dificultades para reintegrarme en el sistema español por falta de reconocimiento de los méritos profesionales adquiridos en el extranjero | O |
| - [3.27] Otros motivos: | O |

1. **Seleccione los 3 principales motivos que le hacen permanecer en el país donde actualmente vive y trabaja en orden de importancia (**en el desplegable solo le aparecerán las opciones que previamente ha seleccionado en la página anterior):

Primer motivo [ ]

Segundo motivo [ ]

Tercer motivo [ ]

1. **¿Consideraría volver a España a trabajar?** Marque **solo una opción** de entre las siguientes:

| O | No, en absoluto |
| --- | --- |
| O | Todavía no, pero me plantearía volver si las condiciones de trabajo de los/as médicos/as de familia en España mejorasen |
| O | Sí, estoy planeando volver principalmente por motivos personales |
| O | Sí, estoy planeando volver principalmente por motivos laborales |

1. ¿**Qué tendría que cambiar** para que se planteara volver a España a trabajar? Marque **tantas opciones como considere**:

|  | **Se corresponde conmigo** |
| --- | --- |
| [7.1.] Oportunidades para investigar, incluyendo mejoras en financiación, departamentos de atención primaria multidisciplinares y/o posibilidad de combinarlo con el trabajo clínico | O |
| [7.2.] Fortalecimiento de los vínculos con facultades de medicina y/o programas de especialidad, incluida la posibilidad de combinar la docencia con el trabajo clínico | O |
| [7.3.] Acceso a una formación médica continuada de alta calidad y oportunidades de especialización adicional | O |
| [7.4.] Oportunidades para involucrarme en la gestión sanitaria a nivel local o regional | O |
| [7.5.] Oportunidades para trabajar en otro ámbito asistencial diferente a la atención primaria (ej., urgencias (extra)hospitalarias, paliativos, etc.) | O |
| [7.6.] Mejor salario y pensión | O |
| [7.7.] Seguridad laboral y económica, con acceso a contratos de larga duración y/o puestos permanentes | O |
| [7.8.] Oportunidades para aumentar y diversificar los ingresos a través responsabilidades adicionales (ej., horario extendido, sustituciones, gestión sanitaria, docencia y/o investigación) | O |
| [7.9.] Oportunidades para compaginar la asistencia en el sistema público y el sector privado | O |
| [7.10.] Tiempo suficiente para los/as pacientes | O |
| [7.11.] Menor carga administrativa | O |
| [7.12.] Horario de trabajo flexible y mayor autonomía sobre mi agenda (ej. para conciliar, combinarlo con la investigación, docencia u otros intereses personales o profesionales) | O |
| [7.13.] Mejor equipamiento médico y tecnológico (incluida la historia clínica electrónica) | O |
| [7.14.] Mayor autonomía a nivel de centro de salud para implementar innovaciones (ej., cambios organizativos, contratación de servicios adicionales) | O |
| [7.15.] Gestión del sistema de atención primaria eficaz, con capacidad de respuesta y comprometida con la implementación de medidas de mejora | O |
| [7.16] Mejor accesibilidad para los/as pacientes | O |
| [7.17] Posibilidad de tener un cupo de pacientes asignado (mayor continuidad asistencial o longitudinalidad) | O |
| [7.18] Mejor coordinación con los servicios de salud comunitarios y/o hospitalarios (incluido urgencias, salud mental, especialistas hospitalarios) | O |
| [7.19] Mejor coordinación con salud pública | O |
| [7.20] Mejor colaboración con el equipo del centro de salud (ej. personal de enfermería, auxiliar, administrativo) | O |
| [7.21] Oportunidades para participar en actividades comunitarias de promoción de la salud | O |
| [7.22] Ambiente laboral inclusivo y respetuoso con la diversidad | O |
| [7.23.] Mayor reconocimiento por parte de los colegas de profesión | O |
| [7.24.] Mayor prestigio social por ser médico/a de familia | O |
| [7.25.] Facilidades para reintegrarme en el sistema español, incluido el reconocimiento de los méritos profesionales adquiridos en el extranjero | O |
| [7.26.] Nada, estoy satisfecho/a con la situación actual de la atención primaria española | O |
| [7.27.] Otro: | O |

1. **Seleccione los 3 principales factores que tendrían que cambiar para que se planteara** volver a España a trabajar **en orden de importancia (**en el desplegable solo le aparecerán las opciones que previamente ha seleccionado):

Primer motivo [ ]

Segundo motivo [ ]

Tercer motivo [ ]

1. Información general. Esta información nos ayudará a poner sus respuestas en perspectiva.

¿Cuántos años tiene?

| Edad: |
| --- |

Género:

| O | Hombre |
| --- | --- |
| O | Mujer |
| O | No binario |

¿Cuál es su estado civil?

| O | Soltero/a |
| --- | --- |
| O | En pareja |
| O | Casado/a o unión civil |
| O | Divorciado/a o separado/a |
| O | Viudo/a |
| O | Otro: |

¿Tiene hijo/a(s) viviendo actualmente con usted?

| O | No |
| --- | --- |
| O | Sí |

¿Cuál es su nacionalidad(es)?

| País(es): |
| --- |

¿En qué país cursó la carrera de Medicina?

| País: |
| --- |

En relación con su formación MIR en Medicina Familiar y Comunitaria (MFyC), por favor especifique:

| Año de finalización: |
| --- |
| Comunidad Autónoma: |

¿Ha realizado alguna estancia en el extranjero? Puede seleccionar más de una respuesta:

| O | No |
| --- | --- |
| O | Sí, durante la carrera de medicina (ej., Erasmus) |
| O | Sí, durante la residencia de MFyC (ej., rotación externa) |
| O | Sí, tras la residencia de MFyC (ej., prácticas profesionales). |

¿Cuándo empezó a considerar la posibilidad de emigrar de España?

| O | Durante la facultad de medicina |
| --- | --- |
| O | Durante la residencia de MFyC |
| O | Después de finalizar la residencia de MFyC |

¿En qué situación se encuentra trabajando actualmente? Puede seleccionar más de una respuesta:

| O | MFyC en Atención Primaria |
| --- | --- |
| O | MFyC en Servicios de Urgencias o Emergencias |
| O | MFyC con interés especial o especialización adicional |
| O | Investigador/a |
| O | Gestión de Servicios Sanitarios |
| O | Tutor/a de médicos residentes |
| O | Tutor/a de estudiantes de medicina |
| O | Otra especialidad diferente a la MFyC |
| O | Otro: |

Díganos, ¿trabaja actualmente en el ámbito asistencial de la atención primaria fuera de España (es decir, atendiendo a pacientes)?

| O | No |
| --- | --- |
| O | Sí |

Desde su experiencia trabajando en (al menos) dos sistemas de atención primaria diferentes, ¿cree que la atención primaria española tiene alguna ventaja con respecto al sistema donde actualmente trabaja?

| O | No |
| --- | --- |
| O | Sí. Por favor, especifique a continuación [pregunta abierta condicionada]: |
|  |  |

¿Qué tipo de contrato tiene actualmente? Puede seleccionar más de una respuesta:

| O | Empleado/a directamente por las autoridades sanitarias competentes o por un centro de salud con contrato público |
| --- | --- |
| O | Socio/a o propietario/a de un centro de salud con contratos públicos con las autoridades sanitarias competentes |
| O | Medico/a independiente prestando servicios de sustitución en el sector público o sin ánimo de lucro (e.g., “locum”) |
| O | Empleado/a por la empresa privada |
| O | Proveedor/a privado en centro independiente (sin contratos con ningún centro de salud ni autoridad sanitaria, veo únicamente pacientes privados independientemente de si mis servicios son reembolsados por seguro médico) |
| O | Otro: |

¿Qué tipo de población atiende?

| O | Urbana (o población con más de 50,000 residentes) |
| --- | --- |
| O | Semiurbana (o población entre 2.000 y 50,000 residentes) |
| O | Rural (o población con menos de 2,000 residentes) |
| O | Otro: |

1. ¿Hay algo más que le gustaría añadir?

| Háganoslo saber: |
| --- |
|  |

Como parte del estudio, nos gustaría realizar grupos focales (virtualmente) con médicos/as de familia españoles que se encuentran actualmente trabajando en el extranjero. La duración aproximada será de 60 minutos, en una fecha y hora que os resulte conveniente. ¿Le gustaría que nos pusiéramos en contacto con usted para darle más información al respecto? En caso afirmativo, marque esta casilla y facilítenos su nombre y dirección de correo electrónico a continuación.

| O | Me gustaría que un investigador se pusiera en contacto conmigo para darme más información sobre una posible participación en un grupo focal. |
| --- | --- |
| Nombre: | |
| Dirección de correo electrónico u otro medio de contacto preferido: | |

**Muchísimas gracias por su tiempo y participación.**

Si conoce a otros/as médico/as de familia que completaron la especialidad en España y que hayan abandonado el país para trabajar en el extranjero, también nos gustaría conocer su opinión. Por favor, reenvíeles el enlace a esta encuesta:

[link to survey]

Puede ponerse en contacto con el quipo investigador dirigiéndose a Sara Calderón-Larrañaga en [s.calderon@qmul.ac.uk](mailto:s.calderon@qmul.ac.uk) si tiene cualquier pregunta o desea más información.

**2. Questionnaire in English:**

Thank you for sharing your experiences and views on general practitioners (GPs) who qualified in Spain and decided to leave the country.

Data shows that the Spanish health system has experienced sustained outflows of doctors since 2010, of which the majority are GPs. However, the reasons why GPs leave the country, what they are doing now, or what the barriers are to their return are not clear. This study, commissioned by Amsterdam UMC health services research group in collaboration with the Organización Médica Colegial (OMC) and Spanish GPs and researchers, aims to answer these questions. We hope the insights gained in this study can improve the working environment for GPs in Spain.

To help with this, we would be very grateful if you could answer the following online survey. It asks about your own reasons for leaving the Spanish primary care system and what barriers there are, if any, to your returning to work in Spain again.

The survey will take **25 minutes** to complete. Your response will be anonymous, although you can voluntarily provide us with your contact information at the end of the survey if you wish to take part in a focus group, part of this same study. Survey data will be analysed by researchers at Amsterdam UMC and reported aggregated. The research has secured ethical approval.

Please, get in touch with Sara Calderón-Larrañaga on [s.calderon@qmul.ac.uk](mailto:s.calderon@qmul.ac.uk) if you have any questions.

1. Filter questions

Did you complete your GP training in Spain?

| O | No [end of survey. Automatic message: we regret to inform you that you are not eligible for this study, as it is only aimed at GPs who trained in Spain and decided to migrate.] |
| --- | --- |
| O | Yes [only if they tick yes they can continue] |

Do you currently work or live abroad?

| O | No [end of survey. Automatic message: we regret to inform you that you are not eligible for this study, as it is only aimed at GPs who currently work or live abroad] |
| --- | --- |
| O | Yes [only if they tick yes they can continue] |

In which country do you currently live/work?

| Country: |
| --- |

In which year did you leave Spain?

| Year: |
| --- |

1. What made you leave Spain? Please, tick **all that apply** and then select **the top three** reasons:

|  | **It applies to me** |
| --- | --- |
| - [2.1.] Personal reasons (e.g. better work opportunities for my partner, learn about a different culture, society, improvement of language proficiency) | O |
| - [2.2.] Lack of opportunities to undertake a PhD or do research (e.g., lack of primary care university departments, research infrastructure and/or funding) | O |
| - [2.3.] Lack of relevant continuing professional development and/or specialisation opportunities | O |
| - [2.4.] Lack of opportunities to get involved in training of medical students or GP trainees | O |
| - [2.5.] Lack of opportunities to get involved in health care management and leadership | O |
| - [2.6.] Lack of opportunities to do clinical work in a setting other than general practice (e.g., in emergency departments, hospital-based work) | O |
| - [2.7.] Insufficient salary or pension | O |
| - [2.8.] Short term contracts and lack of job stability |  |
| - [2.9] Lack of remuneration for additional professional activities (e.g., overtime, research, undergraduate training, GP training or continuing medical education activities) | O |
| - [2.10] Lack of opportunities to combine clinical work in the public system and private sector | O |
| - [2.11] Excessive workload due to lack of personnel | O |
| - [2.12] Excessive bureaucracy and administrative work | O |
| - [2.13] Lack of flexible working hours | O |
| - [2.14] Deficiencies in medical equipment or technology (incl. medical records) | O |
| - [2.15] Lack of autonomy at practice level to implement innovations (e.g., organisational changes, recruitment of additional roles) | O |
| - [2.16] Dissatisfaction with primary health care management | O |
| - [2.17] Patient contact accessibility issues | O |
| - [2.18] Lack of relational continuity of care - [2.19] Poor coordination with community and secondary care (incl. A&E, community mental health, hospital) - [2.20] Poor coordination with public health | O |
| - [2.21] Poor collaboration with colleagues within general practice (e.g. with practice nurses, health care assistants, admin team) | O |
| - [2.22] Lack of opportunities to get involved in community-based health promotion | O |
| - [2.23] Discrimination at the workplace on the basis of gender, sexual identity or orientation, race, religion, disability or other. | O |
| - [2.24] Poor recognition amongst colleagues | O |
| - [2.25] Low social prestige from the public | O |
| - [2.26.] Other reasons: | O |

1. Select the three main reasons why you decided to emigrate from Spain in order of importance (only the options that you have previously selected will appear in the drop down):

First reason [ ]

Second reason [ ]

Third reason [ ]

1. What makes you stay in the country where you currently live and work? Please, tick **all that apply** and then **select the top three** reasons:

|  | **It applies to me** |
| --- | --- |
| - [4.1.] Personal reasons (e.g., better opportunities for children and/or partner, meaningful ties with family, friends and/or society) | O |
| - [4.2.] Adequate research facilities, environment and/or funding | O |
| - [4.3.] Access to high-quality continuing professional development, including opportunities for specialisation | O |
| - [4.4.] Stablished links with medical universities and/or GP training programmes, including the possibility of combining teaching activities with clinical practice | O |
| - [4.5.] Possibility of getting involved in health care management and leadership | O |
| - [4.6.] Possibility of doing clinical work in a setting other than general practice (e.g., in emergency departments, hospital-based work) | O |
| - [4.7.] Better pay and pension | O |
| - [4.8.] Long-term contracts and greater job security | O |
| - [4.9.] Possibility of increasing and diversifying income through additional responsibilities (such as, extended hours, locum, healthcare management, training, teaching and/or research) | O |
| - [4.10.] Possibility of combining clinical work in the public system and private sector | O |
| - [4.11.] Sufficient time for patients | O |
| - [4.12.] Less bureaucracy and administrative work |  |
| - [4.13.] Flexible working hours | O |
| - [4.14.] Up-to-date technology, medical equipment and infrastructure | O |
| - [4.15.] Greater autonomy at practice level to implement innovations (e.g., organisational changes, recruitment of additional roles) | O |
| - [4.16.] Responsive healthcare managers and authorities | O |
| - [4.17.] Increased contact accessibility for patients | O |
| - [4.18.] Increased relational continuity of care | O |
| - [4.19.] Good coordination with community and secondary care (incl. A&E, community mental health, hospital) | O |
| - [4.20.] Good coordination with public health | O |
| - [4.21.] Good collaboration with colleagues within general practice (e.g. with practice nurses, health care assistants, admin team) | O |
| - [4.22.] Opportunities to get involved in community-based health promotion | O |
| - [4.23.] Inclusive and diverse working environments | O |
| - [4.24.] Greater recognition amongst colleagues | O |
| - [4.25.] Greater social prestige for being a GP | O |
| - [4.26] Difficulties to be reintegrated into the Spanish system, due to lack of recognition of professional merits achieved overseas | O |
| - [4.27] Other reasons: | O |

1. Select the **three main reasons for staying in the country where you currently live and work in order of importance** (only the options that you have previously selected will appear in the drop down):

First reason [ ]

Second reason [ ]

Third reason [ ]

1. Would you consider returning to Spain to work? Please, tick **only one** of the following:

| O | Not at all |
| --- | --- |
| O | Not yet, but I would consider going back if working conditions for GPs in Spain improved |
| O | Yes, I am planning to return mainly for personal reasons |
| O | Yes, I am planning to return mainly for work reasons |
|  |  |

1. What would have to change for you to consider returning to Spain to work? Please, tick **all that apply** and then **select the top three** reasons:

|  | **It applies to me** |
| --- | --- |
| [7.1.] Opportunities to do research, including dedicated funding, multidisciplinary primary care departments and/or the possibility of combining research with clinical work | O |
| [7.2.] Better links with medical universities and/or GP training programmes, including the possibility of combining teaching activities with clinical work | O |
| [7.3.] High-quality continuing medical education, including opportunities for specialisation | O |
| [7.4.] Opportunities to become involved in health care management at local or regional level | O |
| [7.5] Opportunities to do clinical work in a setting other than general practice (e.g., in emergency departments, hospital-based work) | O |
| [7.6.] Better pay and pension | O |
| [7.7.] Greater job stability, including long-term contracts and/or permanent positions | O |
| [7.8.] Opportunities to increase and diversify income through additional responsibilities (such as, extended hours, locum, healthcare management, training and/or research) | O |
| [7.9.] Opportunities to combine clinical work in the public system and in private institutions | O |
| [7.10.] Sufficient time for patients | O |
| [7.11.] Less bureaucracy and administrative work | O |
| [7.12.] Flexible working hours and greater autonomy over my schedule | O |
| [7.13.] Up-to-date technology, medical equipment and infrastructure to allow for the provision of high-quality, comprehensive clinical care | O |
| [7.14.] Greater autonomy at practice level to allow for the implementation of innovations | O |
| [7.15.] Effective primary healthcare system management, including responsiveness to front-line workers’ demands and commitment to implement improvement measures as needed | O |
| [7.16.] Increased contact accessibility for patients | O |
| [7.17.] Increased relational continuity of care | O |
| [7.18.] Better coordination with community and secondary care (incl. A&E, community mental health, hospital) | O |
| [7.19.] Better coordination with public health | O |
| [7.20.] Better collaboration with colleagues within general practice (e.g. with practice nurses, health care assistants, admin team) | O |
| [7.21.] Opportunities to get involved in community-based health promotion | O |
| [7.22.] Inclusive and diverse working environments | O |
| [7.23.] Greater recognition amongst colleagues | O |
| [7.24.] Greater social prestige for being a GP | O |
| [7.25.] Opportunities to reintegrate into the Spanish system, including the recognition of professional merits achieved while being abroad | O |
| [7.26] Nothing, I am satisfied with the current situation of Spanish primary care | O |
| [7.26.] Other: | O |

1. Select the **three main factors that would have to change** for you to consider returning to Spain to work **in order of importance** (only the options that you have previously selected will appear in the drop down):

First reason [ ]

Second reason [ ]

Third reason [ ]

1. Background-information. This general information about yourself will help us put your answers into perspective.

How old are you?

| Age: |
| --- |

What is your gender?

| O | Male |
| --- | --- |
| O | Female |
| O | Non-binary |

What is your civil status?

| O | Single |
| --- | --- |
| O | Partnered |
| O | Married or civil union |
| O | Divorced or separated |
| O | Widow/er |
| O | Other: |

Do you have children who live in your household?

| O | No |
| --- | --- |
| O | Yes |

What is your nationality(ies)?

| Country(ies): ________ , _________ |
| --- |

In which country did you complete medical degree?

| Country: |
| --- |

About your GP training in Spain. Please, specify:

| Year of Completion: |
| --- |
| Autonomous Community: |

Have you ever undertaken an international placement?

| O | No |
| --- | --- |
| O | Yes, during my medical degree. |
| O | Yes, during my GP training. |
| O | Yes, after my GP training (e.g., professional placements). |

When did you begin to consider emigrating from Spain?

| O | During medical degree |
| --- | --- |
| O | During GP training |
| O | After finishing my GP training |

In which role(s) are you currently working? Tick more than one if applicable:

| O | Practicing GP in primary care (either salaried, partner or freelance/locum) |
| --- | --- |
| O | Practicing GP in urgent care |
| O | GP with special interest or specialisation |
| O | Researcher |
| O | Health service management |
| O | GP Trainer |
| O | Undergraduate Trainer |
| O | Different specialty |

If you are currently working in general practice, please indicate the following [conditional question]:

From your experience working in (at least) two different primary care systems, does the Spanish system have any advantages over the system where you currently work?

| O | No |
| --- | --- |
| O | Yes. Please, specify [conditional open question]: |
|  |  |

What type of contract do you have? Tick more than one if applicable:

| O | Employed by local, regional, national health authority directly (such as regional directorate of health/department of health) |  |
| --- | --- | --- |
| O | Partner or owner of the practice with public contracts with local, regional or national health authority |  |
| O | Employed by a practice directly within public or non-profit sector |  |
| O | Locum within public or non-profit sector |  |
| O | Employed by a private health company |  |
| O | Private independent provider (without contracts with any practice or health authority, I only see private patients regardless of whether they are reimbursed by insurance) | |
| O | Other | |

What type of practice do you work in?

| O | Urban (or a population with more than 50,000 residents) |
| --- | --- |
| O | Semi-rural (or a population between 2,000 and 50,000 residents) |
| O | Rural (or a population with less than 2,000 residents) |
| O | Other: |

1. Is there anything else that you would like to add?

| Please, let us know: |
| --- |
|  |

We plan to organise some online focus groups with some GPs who left Spanish general practice. These will last about **60 minutes** and would be at a time convenient for you. Would you be happy for us to contact you to give you more information? If so, please tick this box and provide your name and email address below so that we can get in touch with you.

Note that, if you do give your name here, your answers to this questionnaire will still be confidential and there is no obligation to agree to a focus group interview.

| O | I am happy to be contacted with more information about a possible online focus group interview by a researcher. |
| --- | --- |
| Name: | |
| Email address or other preferred contact method: | |

**Many thanks for having taken part in this survey.**

If you know any other GPs who qualified in Spain and left the country to work abroad, we would be very pleased to hear from them as well. Please, forward them the link to this survey:

[link to survey]
