# Supplementary material for: What can we learn from general practitioners who left Spain? A mixed methods international study
Source: Hum Resour Health. 2024 Jan 23;22:9. doi: 10.1186/s12960-023-00888-4 (PMC10804741; doi:10.1186/s12960-023-00888-4)
Supplement: Supplementary file 2 — Additional file 2. Qualitative sample characteristics. [file 12960_2023_888_MOESM2_ESM.docx]

**Additional file 2: Appendix S2. Qualitative sample characteristics**

Table S1. Focus group with GPs working in UK

| **Participant** | **Gender** | **Age** | **Country** | **Role** | **Exit year** |
| --- | --- | --- | --- | --- | --- |
| B1.1 | Female | 30-35 | UK | Salaried GP | >2020 |
| B1.2 | Female | 30-35 | UK | Salaried GP, training fellow | 2015-2020 |
| B1.3 | Female | 40-45 | UK | Salaried GP and clinical lead | 2015-2020 |
| B1.4 | Male | 50-60 | UK | GP partner | <2010 |
| B1.5 | Female | 30-35 | UK | Salaried GP, training fellow | >2020 |

Table S2. Focus group with GPs working in different countries

| **Participant** | **Gender** | **Age** | **Country** | **Role** | **Exit year** |
| --- | --- | --- | --- | --- | --- |
| B2.1 | Male | 35-40 | Ireland | GP partner, locum | 2010-2015 |
| B2.2 | Male | 30-35 | RU | GP partner, locum | >2020 |
| B2.3 | Male | 40-50 | France | GP partner | 2015-2020 |
| B2.4 | Female | 35-40 | Ireland | Salaried GP | >2020 |
| B2.5 | Female | 30-35 | Germany | GP with special interest | 2015-2020 |
| B2.6 | Male | 30-35 | France | Salaried GP, Accident &Emergency | >2020 |
| B2.7 | Female | 30-35 | Canada | Academic GP | >2020 |

Table S3. Focus group with GPs working in different countries

| **Participant** | **Gender** | **Age** | **Country** | **Role** | **Exit year** |
| --- | --- | --- | --- | --- | --- |
| B3.1 | Male | 50-60 | France | GP partner | >2020 |
| B3.2 | Male | 35-40 | Sweden | Salaried GP, GP & UG trainer | >2020 |
| B3.3 | Male | 30-35 | Switzerland | GP with special interest | 2015-2020 |
| B3.4 | Female | 40-45 | Belgium | GP partner, GP & UG trainer | 2010-2015 |

UG undergraduate

Table S4. Focus group with academic GPs

| **Participant** | **Gender** | **Age** | **Country** | **Role** | **Exit year** |
| --- | --- | --- | --- | --- | --- |
| B4.1 | Female | 40-50 | UK | Salaried GP, research fellow | 2015-2020 |
| B4.2 | Female | 40-50 | Luxemburg | Academic GP, PhD | 2010-2015 |
| B4.3 | Male | 30-35 | Australia | Academic GP, PhD | 2015-2020 |
| B4.4 | Female | 35-40 | UK | Academic GP, PhD | 2015-2020 |

Table S5. Interview with GPs working in Sweden

| **Participant** | **Gender** | **Age** | **Country** | **Role** | **Exit year** |
| --- | --- | --- | --- | --- | --- |
| B5.1 | Female | 35-40 | Sweden | Salaried GP, GP & UG trainer | 2015-2020 |
| B5.2 | Female | 30-35 | Sweden | Salaried GP, GP & UG trainer | 2015-2020 |

UG undergraduate

Table S6. Interview with GPs working in research and undergraduate training

| **Participant** | **Gender** | **Age** | **Country** | **Role** | **Exit year** |
| --- | --- | --- | --- | --- | --- |
| B6 | Female | 40-50 | Norway | Academic GP, PhD, UG trainer | <2010 |

UG undergraduate

Table S7. Interview with GPs working in clinic, research, and training

| **Participant** | **Gender** | **Age** | **Country** | **Role** | **Exit year** |
| --- | --- | --- | --- | --- | --- |
| B7 | Female | 40-50 | Sweden | Salaried GP, academic GP, GP & UG trainer | <2010 |

UG undergraduate
