# Supplementary material for: What can we learn from general practitioners who left Spain? A mixed methods international study
Source: Hum Resour Health. 2024 Jan 23;22:9. doi: 10.1186/s12960-023-00888-4 (PMC10804741; doi:10.1186/s12960-023-00888-4)
Supplement: Supplementary file 3 — Additional file 3. Focus group/Semi-structured Interview Script. [file 12960_2023_888_MOESM3_ESM.docx]

**Additional file 3: Appendix S3. Focus group/Semi-structured Interview Script**

Presentation: SCL and AGL

Focus group/interview aim:

The purpose of the focus group/interview is to address our second objective, and we greatly value your participation due to your extensive experience in at least two different healthcare systems – the one in which you received your training and the host system you currently work in. Drawing from your unique experience, we are interested in identifying positive aspects that Spain could learn from.

Focus group norms and implications, including:

- Voluntary participation & free to withdraw at any time.
- Discussion will be audio recorded and transcribed.
- Transcriptions will be anonymised and analysed by the research team.
- Results will be published in medical journals or reports, but participants will not be identifiable.
- Gain verbal consent.

Focus group dynamic:

- Ask them to write their names in MS Teams / Zoom
- Discussion in 3 blocks:

1. Presentation & Introduction
2. Discussion & recommendations
3. Summary & wrap up.

**1^st^ PART. INTRODUCTIONS (20 mins)**

*Tell me about:*

- The city where you work.
- In which year did you leave Spain?
- What is your current job? (Type of contract and roles)
- *Icebreaker*: Identify something you like about the primary care system/your work experience in the [country of destination]

**2^ND^ PART. DISCUSSION & RECOMMENDATIONS TO IMPROVE RETENTION AND RETURN (40 mins)**

- To start with (generic question), what positive elements would highlight from your experience working in [country of destination] general practice?
- Job stability and flexibility – what do you mean by this and what can Spanish general practice learn from [country of destination] in this respect?
- Several comments and answers in the questionnaires allude to better salaries and pensions. Again, to what extent and what recommendations would you make to Spain in this respect?
- Continuous medical education and opportunities for professional development – what has been your experience in the [country of destination] and what can Spanish general practice learn in this respect?
- Healthcare management – what differences do you see between Spain and the [country of destination]in relation to health management and what can Spain learn from the [country of destination] in this regard?
- Some comments and answers in the questionnaires also refer to the higher prestige and recognition of the Family Medicine Speciality in [country of destination]. What do you think this is due to, what does it mean, and again, what can Spain learn in this respect?
- Several of you are involved in training residents and medical students, others in research, what has been your experience as trainers, teachers, researchers (as well as clinicians) and what can Spain learn from this?

**3^RD^ PART. SUMMARY & WRAP UP**

- Brief summary.
- Is there anything you’d like to add, or any relevant topic that we did not address, and you’d like to mention.
- Interviewees were notified that they would be informed of the preliminary results of the study and future dissemination channels.
