# Supplementary material for: What can we learn from general practitioners who left Spain? A mixed methods international study
Source: Hum Resour Health. 2024 Jan 23;22:9. doi: 10.1186/s12960-023-00888-4 (PMC10804741; doi:10.1186/s12960-023-00888-4)
Supplement: Supplementary file 4 — Additional file 4. Survey results. [file 12960_2023_888_MOESM4_ESM.docx]

**Additional file 4: Appendix S4. Survey results**

Table S1. Respondents’ professional role and employment

| **Professional role (%)** | Practicing GP in primary care | 108 (68.35) |
| --- | --- | --- |
|  | Practicing GP in urgent care | 19 (12.03) |
|  | GP with special interest | 18 (11.39) |
|  | Undergraduate Trainer | 18 (11.39) |
|  | GP Trainer | 17 (10.76) |
|  | Researcher | 14 (8.86) |
|  | Different specialty | 13 (8.23) |
|  | Health service management | 12 (7.59) |
|  | >1 role | 45 (28.48) |

Table S2. Respondents’ intention to return

| **Would you consider returning? (%)** | Yes, if working conditions in Spain improved | 77 (48.73) |
| --- | --- | --- |
|  | Not at all | 45 (28.48) |
|  | Yes, I’m planning to return | 27 (17.09) |
|  | Missing | 9 (5.7) |

Table S3. Association between intention to return to Spain and respondents’ sociodemographic characteristics

|  | **INTENTION TO RETURN** | |
| --- | --- | --- |
|  | **OR (95% CI)** | **OR (95% CI)*** |
| **Gender** |  |  |
| Male | 1 | 1 |
| Female | 2.04 (0.96-4.35) | 1.95 (0.87-4.38) |
| **Age** |  |  |
|  | 0.97 (0.93-1.01) | 0.98 (0.93-1.03) |
| **Nationality** |  |  |
| Spanish | 1.06 (0.28-4.02) | 0.87 (0.21-3.61) |
| Mixed Spanish | 2.22 (0.36-13.54) | 2.09 (0.30-14.42) |
| Other | 1 | 1 |
| **Country** |  |  |
| UK | 3.27 (0.99-10.77) | 5.92 (1.52-22.97) |
| France | 0.63 (0.21-1.88) | 0.79 (0.24-2.62) |
| Ireland | 1.47 (0.46-4.73) | 1.50 (0.42-5.40) |
| Sweden | 1.73 (0.46-6.47) | 1.64 (0.41-6.57) |
| Other | 1 | 1 |
| **Exit year** |  |  |
| Pre2015 | 1 | 1 |
| Post2015 | 1.29 (0.52-3.20) | 1.93 (0.62-6.04) |

OR comparing those who would return if working conditions in Spain improved with those who would not return (baseline category); *OR adjusted by the remaining socio-demographic variables included in the table.
